# Red Blood Cell Distribution Width in Heart Failure: Pathophysiology, Prognostic Role, Controversies and Dilemmas

**DOI:** 10.3390/jcm11071951

**Published:** 2022-03-31

**Authors:** Andrew Xanthopoulos, Grigorios Giamouzis, Apostolos Dimos, Evangelia Skoularigki, Randall C. Starling, John Skoularigis, Filippos Triposkiadis

**Affiliations:** 1 Department of Cardiology, University Hospital of Larissa, 41110 Larissa, Greece; andrewvxanth@gmail.com (A.X.); grgiamouzis@gmail.com (G.G.); dimos_ap@hotmail.com (A.D.); evangelia.skoul@gmail.com (E.S.); iskoular@gmail.com (J.S.); 2 Kaufman Center for Heart Failure and Recovery, Heart, Vascular, and Thoracic Institute, Cleveland Clinic, Cleveland, OH 44195, USA; starlir@ccf.org

**Keywords:** red blood cell distribution width, heart failure, prognosis, mechanisms

## Abstract

Red blood cell distribution width (RDW), an integral parameter of the complete blood count (CBC), has been traditionally used for the classification of several types of anemia. However, over the last decade RDW has been associated with outcome in patients with several cardiovascular diseases including heart failure. The role of RDW in acute, chronic and advanced heart failure is the focus of the present work. Several pathophysiological mechanisms of RDW’s increase in heart failure have been proposed (i.e., inflammation, oxidative stress, adrenergic stimulation, undernutrition, ineffective erythropoiesis, reduced iron mobilization, etc.); however, the exact mechanism remains unknown. Although high RDW values at admission and discharge have been associated with adverse prognosis in hospitalized heart failure patients, the prognostic role of in-hospital RDW changes (ΔRDW) remains debatable. RDW has been incorporated in recent heart failure prognostic models. Utilizing RDW as a treatment target in heart failure may be a promising area of research.

## 1. Introduction

**Figure 1:**
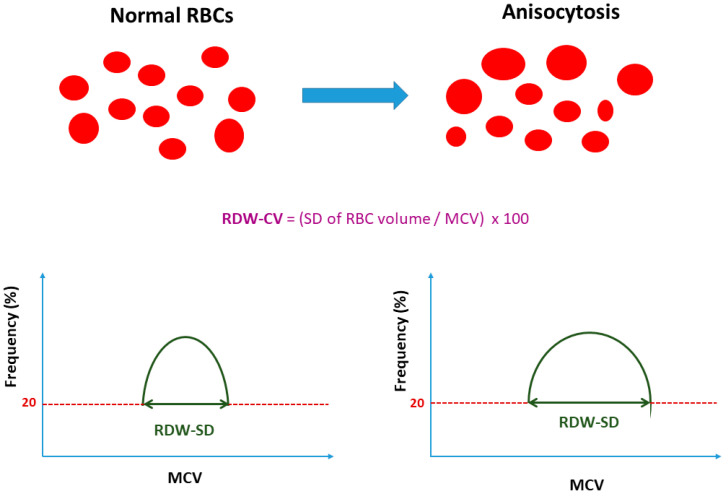
Definition and calculation of the red blood cell distribution width (RDW); in the case of anisocytosis, RDW increases. RDW-CV, coefficient of variation of erythrocyte volumes; RDW-SD, standard deviation of erythrocyte volumes; RBC, red blood cell; MCV, mean corpuscular volume.

Red blood cell distribution width (RDW) is an integral parameter of the complete blood count (CBC), which has been traditionally used for the classification of several types of anemia [1]. It has been defined either as the standard deviation (SD) of erythrocyte volumes (RDW-SD), which is measured by calculating the width at the 20% height level of the red blood cell (RBC) size distribution histogram; or, as the coefficient of variation (RDW-CV) of erythrocyte volumes by dividing the standard deviation (SD) of the red blood cell volume (RBCs) by the mean corpuscular volume (MCV) multiplied by 100 (SD/MCV × 100), and expressing the variability in size of circulating erythrocytes (anisocytosis) (Figure 1) [2]. The normal reference ranges of RDW-SD and RDW-CV are typically 39–46 fL and 11.5–15%, respectively, but often vary depending on the method of RDW calculation and the available hematological analyzers used [3].

Over the last decade, changes in RDW have been associated with outcomes in several discrete populations [4,5], such as in patients with cardiovascular disease [6] including stable coronary artery disease [7], acute coronary syndromes [8], acute myocardial infarction [9], stroke [10] and heart failure (HF). The changes of RDW in HF is the focus of the present work. In this regard, the prevalence, direction and clinical significance of RDW changes, and the contribution of RDW changes to outcome prediction in the relevant HF prognostic models are summarized. Further, the potential underlying mechanisms of RDW changes in acute, chronic and advanced HF as well as the areas of future research regarding this hematological marker will be discussed.

## 2. (Patho)Physiology of RDW Increase in Heart Failure

Erythropoiesis mainly occurs in the bone marrow [11]. Traditionally, it is considered that the main physiological role of RBCs, or erythrocytes, is to transport oxygen and carbon dioxide from the lung to the tissues and to maintain systemic acid/base equilibria [12]. However, recent clinical and experimental evidence suggests that RBCs may be directly involved in nitric oxide (NO) metabolism and control of blood rheology, as well as erythrocrine function (i.e., by releasing nitric oxide (NO), NO metabolites and adenosine triphosphate) [12]. In humans, the life span of RBCs is 120 days. Under normal conditions, approximately 1% of RBCs are synthesized each day, but RBC production can increase substantially during times of acute or chronic stress [11]. The typical mature erythrocyte is disc-shaped, with a diameter between 6–8 μm and a total volume, also known as mean corpuscular volume (MCV), ranging between 80 and 100 fL [13]. Nevertheless, the mechanical properties and lipid architecture are altered in RBCs from subjects with cardiovascular risk factors, resulting in reduced tissue perfusion, increased oxidative stress, reduced oxygen delivery and an increase in whole blood viscosity which may lead to increased cardiovascular mortality [14].

**Figure 2:**
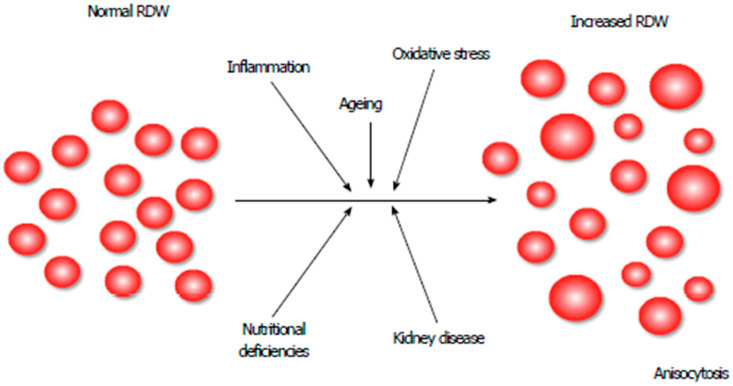
Pathophysiological mechanisms causing anisocytosis. RDW: red blood cell distribution width [13].

The pathophysiological mechanism of RDW’s rise (i.e., increased anisocytosis) in HF remains obscure (Figure 2) [13]. It has been suggested that inflammation, neurohormonal and adrenergic system activation may bring about changes in RBC maturation by disturbing the red cell membrane, thereby leading to increased RDW [15]. HF is associated with the activation of both cell- and cytokine-mediated inflammatory pathways, which can impair bone marrow function with the release of premature erythrocytes into the circulation [16]. A correlation between RDW and established inflammatory markers such as interleukin-6 (IL-6) and C-reactive protein (CRP) has been reported [17,18].

The nervous system emerges as a critical regulatory player of the bone marrow; the primary site of postnatal hematopoiesis and hematopoietic stem cell (HSC) maintenance [19]. Several studies support the central role of the sympathetic nervous system (SNS) in the regulation of hematopoiesis [20]. Norepinephrine is delivered to the bone marrow by the sympathetic nerve in a circadian (diurnal) manner which regulates the expression of core genes in the bone marrow, leading to the fluctuation of immune cell egress in both humans and rodents [21]. A correlation between the proliferation of many HSCs and the circadian oscillation in concentrations of norepinephrine in the bone marrow has also been reported [22]. Therefore, a close communication exists between the SNS and the bone marrow, and dysregulation in this communication may lead to aberrant hematopoietic and immune system responses and increased RDW values [23]. Ineffective erythropoiesis, reduced iron mobilization, oxidative stress, renal failure as well as nutritional deficiencies have been also implicated in the pathophysiology of RDW increase in HF patients [17,18].

## 3. RDW Change in Heart Failure

### 3.1. RDW in Incident Heart Failure

Several studies have demonstrated that RDW is an independent predictor of incident HF. In a retrospective population-based cohort analysis including 26,784 middle-aged subjects without history of myocardial infarction, stroke or HF, who participated in the Malmo Diet and Cancer study (1991–1996) and were followed up for up to 15 years, a higher RDW was found to be associated with higher long-term incidence of first hospitalization due to HF (Hazard Ratio (HR): 1.47, 95% Confidence Interval (CI): 1.14–1.89, in the top compared with the bottom quartile of RDW), after adjusting for history of coronary revascularization, biological, lifestyle and socio-economic factors [24]. Emans et al. reported that RDW levels were associated with an increased risk of incidence of HF utilizing data from 17,533 participants from the European Prospective Investigation into Cancer and Nutrition (EPIC)-Norfolk cohort [25]. Among them, 640 participants developed HF during a follow-up of 11.2 ± 2.2 years and authors observed that there was a non-linear increase in HF risk as RDW values were increasing [25].

### 3.2. RDW in Chronic HF

The RDW is associated with indices of cardiac function and/or disease severity in HF including the natriuretic peptides [26,27], peak oxygen consumption [28,29], left ventricular end diastolic pressure (LVEDP) [30] and left ventricular deformation [31,32].

The first study examining the predictive value of RDW in chronic HF was conducted by Felker et al. back in 2007 [33]. The authors found that RDW exhibited the strongest association with cardiovascular death or HF hospitalization (adjusted HR 1.17 per 1-SD increase, 95% CI: 1.10–1.25, *p* < 0.001) among 36 laboratory parameters in a cohort of 2679 symptomatic chronic HF patients from the North American CHARM (Candesartan in Heart Failure: Assessment of Reduction in Mortality and Morbidity) program. Similar were the findings in the Study of Anemia in a Heart Failure Population (STAMINA-HFP) [18,34], as well as in patients post myocardial infarction (MI) [35]. In this regard, it has been proposed that RDW may be a better predictor of outcomes in HF patients than several echocardiographic parameters [36]. It is noteworthy that no association was observed between RDW and HF due to valvular heart disease [37]. A recent study reported an independent association between a low hemoglobin/RDW ratio and the risk of death as well as the combined endpoint of death or cardiovascular hospitalization in a cohort of 6888 HF patients. Interestingly, this association was observed over the whole spectrum of HF types, including HFrEF and HFpEF [38].

### 3.3. RDW in Acute HF

Increased RDW may be associated with a slower diuretic response [39], and elevated LV filling pressure (E/E’) [40], and has been shown to predict early mortality in patients presenting with acute dyspnea at the emergency department (ED), irrespective of its etiology [41]. Notably, the addition of RDW to conventional laboratory tests (B-type natriuretic peptide, creatinine, sodium and chloride) may significantly improve the 30-day prognostic assessment (for all-cause mortality) of acute HF patients presenting in the ED [42].

The prognostic value of RDW in acute HF was initially reported in a Spanish study, which included 628 consecutive patients [43]. Pascual-Figal et al. collected clinical, echocardiographic and laboratory variables at discharge and followed up with patients for ~2½ years. RDW was found to be a strong and independent marker of all-cause mortality (*p* = 0.004, HR 1.072, 95% CI 1.023–1.124), regardless of anemia status (*p* for interaction > 0.1 for the entire population). Therefore, this study demonstrated that RDW may be used as an early predictor of adverse outcomes in non-anemic HF patients. Subsequently, it was demonstrated that RDW provided additional prognostic information to natriuretic peptides [44,45,46,47,48], that it may also be useful in the elderly [49] and that it may be a better predictor of outcome in patients with a preserved, than in those with reduced, left ventricular ejection fraction (LVEF) [50,51]. Patients with high RDW and N-terminal pro b-type natriuretic peptide (NT-proBNP) values exhibit the highest mortality rates, whereas those with low RDW and NT-proBNP exhibit the most favorable outcomes. Finally, a significant interaction between diabetes and RDW longitudinal changes from the patient’s admission to 1-year post discharge has been reported [52].

### 3.4. RDW in Advanced HF

A retrospective analysis of 367 consecutive patients with advanced HF (New York Heart Association class III-IV) and concomitant diabetes mellitus revealed RDW as an independent predictor of all-cause mortality during the long term follow up [53].

RDW may be useful in risk stratification of patients selected for implantable cardioverter defibrillator (ICD) therapy as it predicts death and appropriate therapy [54]. In patients with advanced HF undergoing cardiac resynchronization therapy (CRT), it has been reported that elevated RDW is associated with lower rates of “positive response” to CRT (defined as a reduction in LV end-systolic volume (LVESV) ≥15% or a relative increase in LVEF ≥ 15%) [55,56], and that elevated the RDW level before and after CRT implantation is independently associated with all-cause mortality [57].

A retrospective single center analysis of 188 continuous flow left ventricular assist devices (LVADs) implanted from 2004 to June 2014, revealed an independent association between higher pre-implant RDW values and mortality in a more than 1 year follow up [58]. Similarly, in the study by Truby et al. RDW was independently predictive of 90-day mortality (Odds Ratio (OR), 1.16 for 1% increase; CI, 1.04–1.31; *p* = 0.010) in a population of 409 continuous-flow LVADs [59]. Notably, mechanical unloading with continuous-flow LVADs was associated with a reduction in RDW levels. On the contrary, Ahmad et al. reported no change in RDW values, at a median of 135 days after LVAD placement [60]. Poglajen et al. proceeded to a 5-day bone narrow stimulation with granulocyte colony stimulating factor (G-CSF) in 44 patients with advanced HF [61]. On the fifth day, a full blood count and a peripheral blood CD34+ cell count were performed. On a multivariable analysis, RDW turned out to be a sole independent predictor of poor stem cell mobilization (HR 8.64, 95% CI: 1.242–60.021, *p* = 0.01). Although the underlying mechanisms are not clearly defined, it appears that RDW may represent a useful tool to improve the selection of candidates for stem cell therapy in this population of patients [61]. Lastly, increased RDW in advanced HF patients immediately before orthotopic heart transplantation is an independent predictor of post-operative mortality during an average follow-up of 45.5 months [62].

## 4. RDW Longitudinal Changes in HF

### 4.1. Acute HF

Several studies have reported the association between an increase in RDW longitudinal values (ΔRDW) during HF hospitalization and adverse short [63,64] as well as long-term outcomes [65,66,67]. Enlarging RDW during hospitalization has added valuable prognostic information on top of hemoconcentration [68], whereas patients with positive ΔRDW between admission and 1 month after discharge have demonstrated a significantly higher cardiovascular mortality and HF re-hospitalizations compared with those with no positive RDW changes [69]. Núñez et al. reported the longitudinal association of RDW with mortality and anemia in 1702 patients recently hospitalized for AHF [70]. The investigators utilizing sophisticated statistical methods found that the RDW trajectory differs according to the patient’s outcome, with relatively stable RDW levels over time for those patients who remained alive during the follow up (median follow-up = 1.5 years), and with a steeper slope near its occurrence for those who died [70].

### 4.2. Chronic HF

In a cohort of 6159 ambulatory chronic HF patients, RDW’s positive change from baseline to 1-year follow up was associated with all-cause mortality over a mean period of 4.4 ± 2.4 years (risk ratio for +1% increase in changes in RDW was 1.08 (95% CI 1.03–1.13; *p* = 0.001)) [71]. This effect was independent of anemia status or other baseline cardiac or renal indices, and particularly strong in those with a lower baseline RDW. In another study of 274 chronic HF patients, the combination of expanding RDW value and the evolving iron deficiency (falling MCV) over time conferred a three-fold escalated risk of mortality independently of changes in hemoglobin and RDW [72].

Taken all together, the above studies suggest that monitoring changes in RDW may serve as a robust longitudinal indicator for outcomes in the entire HF spectrum. Nevertheless, these findings were not replicated in a recent study which analyzed data from two cohorts with AHF patients [73]. In particular, the ΔRDW (admission RDW—discharge RDW) was similar between patients who developed [0.2 (−0.2 to 0.7)] and those who did not develop [0.0 (−0.1 to 0.5)] the study endpoint all-cause death or HF rehospitalization at 1 year (*p* not adjusted for ties = 0.863; *p* adjusted for ties = 0.271). The contradictory findings between the latter study and previous studies indicating that ΔRDW during hospitalization has clinical significance are incompletely understood. A potential explanation might be that the value of ΔRDW is small and may lie within the laboratory (assay) variation. Moreover, there is compelling evidence indicating that RDW may reflect inflammation [74]. Interestingly, inflammatory markers remain practically unaffected in the short term in hospitalized HF patients [75]. Nevertheless, whether RDW is the glycated hemoglobin (HbA1c) of the HF patient or not needs to be answered in future studies.

## 5. Systematic Reviews and Meta-Analyses

A systematic review and meta-analysis of 17 cohort studies with a total of 18,288 patients hospitalized for HF showed that RDW on admission and RDW at discharge, as well as its change during treatment, were of prognostic significance for HF patients [76]. In particular, a 1% increase in baseline RDW was associated with a 10% higher risk of all-cause mortality [76]. Similarly, in the meta-analysis by Shao Q et al. (17 studies and 41,311 patients), for every 1% increase in RDW, the risk of all-cause mortality increased by 12% (HR 1.12, 95% CI 1.08–1.16) [77]. In addition, every 1% increase in RDW was associated with a 9.1% increase in the risk of HF hospitalization (HR 1.091, 95% CI 1.025–1.162, I2 = 52.8%). Lastly, the meta-analysis by Hou H et al. consisting of 28 studies and 102,689 participants, revealed an HR of 1.12 (95% CI  =  1.09–1.15) for all-cause mortality and an HR of 1.12 (95% CI  =  1.08–1.17) for major adverse cardiac events, per 1% increase of RDW [78].

## 6. RDW and Prognostic Scores in HF

Risk prediction in HF provides information about patient prognosis, guides decision making and enables better understanding of provider performance [79]. A number of prognostic models have been developed both in acute and chronic HF [80,81]. RDW has been incorporated in some of them. For example, the Larissa Heart Failure Risk Score (LHFRS) is a simple prognostic model consisting of three variables, namely, history of hypertension, history of coronary artery disease/myocardial infarction and RDW ≥ 15% [82,83]. Recently, this score has been externally validated in a cohort of 1670 consecutive AHF patients enrolled in The Registry Focused on Very Early Presentation and Treatment in Emergency Department of Acute Heart Failure (REALITY-AHF) [84] and found to be an independent predictor of 1-year all cause-death or HF rehospitalization (adjusted HR per 1-point increase, 95%CI: 1.17 [1.04–1.32], *p* = 0.011) [84].

A risk score derived from the machine learning assessment of risk and early mortality in heart failure (MARKER-HF) model accurately discriminated between low and high-risk of death (AUC = 0.88) by identifying eight variables, including RDW [85]. Lastly, RDW was included in the sex-specific intermountain inpatient heart failure model (iHF) for males [86]. The latter was derived during an inpatient admission (within 24 h of hospitalization) and effectively predicted post-discharge 30-day readmission in independent validation populations.

## 7. RDW and Congenital Heart Disease

Congenital heart disease (CHD) is a common cause of HF and several studies have examined the association between RDW and outcomes in CHD patients. Increased RDW is a predictor of worse outcome in children undergoing surgery for CHD [87], in children with HF due to CHD [88] and in children with Tetralogy of Fallot undergoing corrective repair [89]. Interestingly, RDW is associated with central venous pressure and SVO2 in pediatric patients with Fontan circulation [90]. In adult patients with CHD, elevated RDW has been associated with increased IL-6 [91], low exercise capacity [92], as well as adverse cardiovascular events and poor survival [93,94,95].

## 8. RDW and Anemia

Nutritional deficiencies and heterozygous hemoglobinopathies are present in many forms of anemia and are characterized by different degrees of anisocytosis [13]. However, several studies in the general population [5,24,25], as well as in acute [43,46,66,70], chronic [33,96] and advanced [97] heart failure have demonstrated the independent (of anemia) prognostic value of RDW. Nevertheless, keeping in mind that RDW is strongly inversely associated with anemia, its interpretation in anemic patients should be made with caution.

## 9. Future Perspectives

A fruitful area of research may be the evaluation of the prognostic value of RDW in cardio-oncology [98]. Until now, RDW has been used mainly for the assessment of prognosis, either as a sole marker or as a part of risk models, in HF patients. Nevertheless, keeping in mind that RDW reflects several mechanisms of HF development may expand its indications from a marker of prognosis to a treatment target (Table 1). Interestingly, data from the BIOSTAT-CHF (Biology Study to Tailored Treatment in Chronic Heart Failure) showed that up-titrating patients with HF based on biomarker values might have resulted in fewer deaths or hospitalizations compared with a hypothetical scenario in which all patients were successfully up-titrated [99]. Lastly, mechanistic studies regarding RDW increase in HF would be of great interest.

## 10. Conclusions

**Figure 3:**
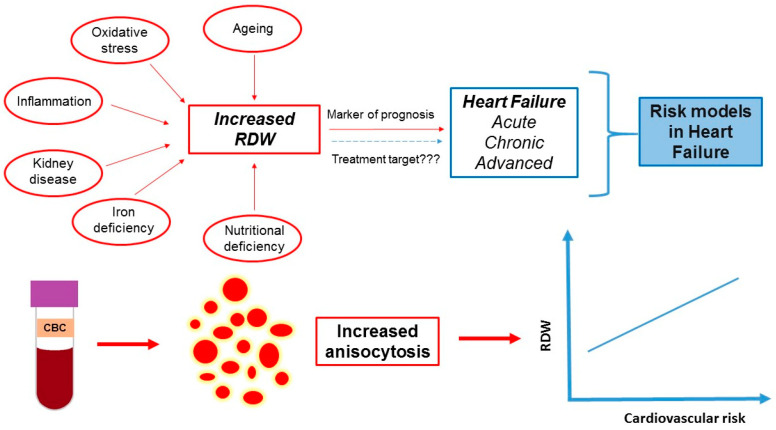
The role of RDW in heart failure. RDW is a simple parameter derived from the complete blood count (CBC) and expresses the variability in red blood cell size (anisocytosis). RDW has been used as a marker of prognosis in acute, chronic and advanced heart failure either as a sole variable or as a variable included in risk models. Higher RDW has been associated with increased risk of death and/or re-hospitalization. Several (patho)physiological mechanisms such as ageing, oxidative stress, inflammation, kidney disease, iron deficiency and nutritional deficiency have been implicated in the reported RDW increase in patients with heart failure. Whether or not RDW can be used as a treatment target remains to be elucidated in future studies.

RDW is a simple marker of the CBC initially used for the classification of different types of anemia. It has an established prognostic role in acute, chronic and advanced HF (Table 2, Figure 3). A controversy exists regarding the prognostic value of in-hospital RDW changes in acute HF. Utilizing RDW as a treatment target in HF may be a promising area of research.

## Figures and Tables

**Table 1:** Facts and open issues about RDW.

Facts	Open Issues/Controversies
RDW is an integral marker of the complete blood count and can be calculated by automatic hematology analyzers.	No universal consensus on the recommended method of RDW calculation (standard deviation or coefficient of variation) currently exists.
The typical reference range value of RDW-CV is 11.5–15% and of RDW-SD is 39–46 fL.	No universal reference range values exist. Those often vary depending on the method of RDW calculation and the available hematological analyzers used.
RDW is an established simple prognostic marker in heart failure (acute, chronic and advanced).	There are limited data on the role of RDW in cardio-oncology.
Several pathophysiological mechanisms of the RDW increase in heart failure have been proposed (inflammation, adrenergic stimulation, undernutrition, etc.).	The exact pathophysiological mechanism of RDW increase in heart failure remains unknown.
RDW values at hospital admission and discharge have been associated with prognosis in heart failure patients.	There is a debate on the prognostic value of in-hospital RDW changes (ΔRDW).
The current RDW indications include the classification of several types of anemia and the estimation of patients’ risk in cardiovascular diseases (including heart failure)	RDW may be used in the future to guide therapy in heart failure.

**Table 2:** Studies examining the prognostic value of RDW in (a) the general population, (b) chronic, (c) acute and (d) advanced heart failure.

Reference	Number of Subjects	Study Design	Outcome	Results	Conclusion
**(a) Population-Based Cohort**					
[25]	17,533	Retrospective (Mean follow up 11.2 years)	Incident HF	Adj. HR 1.44, (95% CI 1.15–1.80)	RDW is associated with HF events in an apparently healthy middle-aged population.
[24]	26, 784	Retrospective (Mean follow up 15 years)	Risk of hospitalization due to HF	Adj. HR 1.47, (95% CI 1.14–1.89)	Red cell distribution width was associated with long-term incidence of first hospitalization due to HF among middle-aged subjects.
**(b) Chronic Heart Failure**					
[38]	6888	Retrospective (Follow up 24 months)	All-cause mortality and cardiovascular hospitalization	A lower Hb/RDW ratio was a predictor of mortality (Q1 vs. Q6: Adj HR 1.84 (1.63–2.08)	Hb/RDW ratio is a prognostic tool for predicting HF mortality and cardiovascular hospitalizations.
[32]	169 HFpEF vs. 50 controls	Prospective	Predictive value of deformation imaging combined with RDW	The associations of clinical and echocardiographic parameters with HFpEF were improved by adding RDW (*p* < 0.01)	RDW has an independent incremental predictive value for HFpEF.
[31]	59 HFrEF vs. 40 controls	Prospective	LV global longitudinal strain	RDW showed negative correlations with LV global longitudinal strain (r = −0.41, *p* = 0.001)	Elevated RDW is associated with poorer LV deformation assessed by speckle tracking echocardiography in HF patients with similar EF.
[30]	1084	Prospective	LVEDP, mortality	RDW was independently associated with high LVEDP (Adj. OR per unit change 1.14, 95% CI 1.0 to 1.29) and 5 year-mortality (Adj. HR 4.11, 95% CI 2.12 to 7.96)	RDW was independently associated with high LVEDP and with mortality.
[36]	232	Prospective (Follow up 12 months)	Cardiovascular death and/or HF hospitalization	RDW > 14.45%, Adj. OR:3.894, (95%CI 1.042–14.55)	RDW is a better predictor of adverse outcome than several echocardiographic parameters.
[27]	215	Prospective (Mean follow up 24.2 months)	All-cause mortality	Adj. OR: 2.963 (95% CI 1.066–6.809)	RDW may be an indicator in the risk stratification.
[35]	350	Retrospective (Follow up 12 months)	All-cause mortality and HF hospitalization	Higher mortality and HF re-admission in patients with RDW > 14.5 (vs. RDW ≤ 14.5) (*p* < 0.001 and *p* = 0.004, respectively). Levels of RDW were associated with the presence of severe LV dysfunction (LVEF < 30%) *	Elevated RDW may be used as a prognostic tool among HF patients with the documented myocardial infarction.
[34]	165	Prospective (Follow up 24 months)	All-cause mortality	Adj. HR 1.19 (95% CI 1.004–1.411) at 12 months	RDW is an independent predictor of mortality at 12 months, but it loses its significance during longer-term follow up.
[37]	1021 (CHD vs. DCM vs. VHD)	Retrospective (Mean follow up 21 months)	All-cause mortality	The AUC of RDW for predicting mortality due to CHD and DCM was 0.704 (95% CI 0.609–0.799) and 0.753 (95% CI 0.647–0.860), respectively. The AUC of the RDW for predicting mortality from VHD was 0.593	RDW is a prognostic indicator for patients with HF caused by CHD and DCM.
[29]	85 HF vs. 107 controls	Prospective	Peak VO2, VE/VCO2 slope	RDW is an independent predictor for peak VO2 (β = −0.247, *p* = 0.035) and VE/VCO2 slope (β = 0.366, *p* = 0.004)	Higher RDW is independently related to peak VO2 and VE/VCO2 slope.
[28]	118	Prospective	Exercise capacity	Log[RDW] is associated with VO2peak (β = –0.277, *p* = 0.003)	Higher RDW is independently related to impaired exercise capacity.
[96]	698	Prospective (Median follow up 2.5 years)	All-cause mortality HF hospitalization	All-cause mortality HR (for RDW > 15.4%) 2.63, (95% CI 2.01–3.45) HF hospitalization HR (for RDW > 15.4%) 2.37, (95% CI, 1.80–3.13)	RDW value is a risk marker for the occurrence of both death and hospitalization for HF in outpatients with chronic HF, independent of anemia.
[26]	1087	Retrospective (Median follow up 52 months)	All-cause mortality	Adj. HR 1.12, (95% CI 1.05–1.16)	RDW has similar independent prognostic power to NT-proBNP.
[33]	2679	Retrospective (Median follow up 34 months)	Morbidity and mortality	Adj. HR 1.17 per 1-SD increase, *p* < 0.001	RDW is an independent predictor of morbidity and mortality.
**(c) Acute Heart Failure**					
[49]	897 (≥65 years)	Retrospective	All-cause mortality at 1 year	Adj. HR 1.41 (95% CI, 1.05–1.90)	A higher baseline RDW was associated with increased risk for 1-year all-cause mortality.
[42]	2278 ED visits	Retrospective (Follow up 4 years)	All-cause mortality at 30 days	AUC 0.723, (95% CI 0.693–0.763)	The prognostic assessment of acute HF patients in the ED can be improved by combining RDW with other laboratory tests.
[52]	218 patients (71 diabetics)	Prospective (Follow up 1 year)	All-cause mortality or rehospitalization for HF at 1 year	Diabetics: Adj HR: 1.349, (95% CI 1.120–1.624) Non-diabetics: Adj HR: 1.142, (95% CI 1.011–1.291 (βinteraction = −0.002; SE = 0.001; *p* = 0.042) between DM and RDW longitudinal changes	RDW has similar prognostic significance (diabetic and non-diabetic) in HF patients. RDW longitudinal changes show significant difference in diabetic and non-diabetic patients.
[51]	278 HFpEF patients	Retrospective (Follow up 3 years)	Non cardiac mortality	Adj. HR 1.169, (95% CI 1.042–1.311)	RDW levels at admission independently predict non-cardiac mortality in acute HFpEF.
[50]	402	Prospective	All-cause mortality at 1 year	All-cause mortality of all patients increased with quartiles of rising RDW (χ^2^ 18; *p* < 0.001).	High RDW predicts mortality in acute HF.
[47]	128	Prospective (Follow up 3 months)	Cardiac death and/or readmission for HF	Adj. HR 4.610, (95% CI 1.935–10.981)	RDW and NT-proBNP are independent predictors of 90-day cardiovascular events in patients hospitalized with HF. RDW adds prognostic value to NT-proBNP.
[46]	521	Prospective (Median follow up 24 months)	In-hospital mortality, All-cause mortality and HF readmission (long term)	In-hospital mortality (for log RDW): coef. 5.21, *p* = 0.044, All-cause mortality and HF re-admission (long term): RDW (per SD increase, HR 2.19; 95% CI 1.92–2.50; *p* < 0.0001)	Higher RDW values in acute HF at admission are associated with worse short- and long-term outcomes and RDW values are more prognostically relevant than hemoglobin levels.
[39]	100	Retrospective	Slow diuretic response	Adj. OR 1.47, (95 % CI 1.07–2.02)	High RDW at admission is a predictor of slower diuretic response.
[41]	907	Retrospective	All-cause mortality at 30 days	Adj.HR 1.23, (95% CI 1.11–1.36)	RDW measured at ED is an independent predictor of early mortality.
[45]	789	Retrospective (Median follow up 573 days)	All-cause mortality	Adj. HR 3.21, (95% CI 1.77–5.83)	Discharge RDW is an independent predictor of all-cause mortality in predominantly African American patients.
[48]	205	Retrospective (Follow-up 1 year)	All-cause mortality	Adj. HR = 1.03 per 1% increase in RDW, (95% CI 1.02–1.07, *p* = 0.04)	RDW independently predicted 1-year mortality in acute HF.
[43]	628	Prospective (Median follow up 38.1 months)	All-cause mortality	Adj. HR 1.072, (95% CI 1.023–1.124)	Higher RDW levels at discharge are associated with a worse long-term outcome, irrespective of hemoglobin levels.
[44]	707	Prospective (Median follow up 421 days)	All-cause mortality	Adj. HR 1.06, (95% CI 1.01–1.11)	RDW provides incremental prognostic value to BNP in acute heart failure. The prognostic ability of RDW is independent of hemoglobin concentration.
[40]	100	Prospective	Relation between RDW and echocardiographic parameters	RDW was independently correlated with E/E (β-coefficient 0.431, *p* = 0.001)	RDW may be associated with elevated LV filling pressures in patients with acute HF.
**(d) Advanced Heart Failure**					
[59]	409 patients with cf-LVADs	Retrospective	All-cause mortality at 90 days	Adj. OR 1.16 for 1% increase, (95% CI: 1.04–1.31)	RDW is an independent predictor of 90-days mortality in cf-LVAD patients.
[53]	367	Retrospective (Mean follow up 4.4 years)	All-cause mortality	Adj. HR 1.0492 (95 % CI 1.0247–1.0743)	RDW is an independent predictor of all-cause mortality in advanced HF patients with concomitant diabetes mellitus.
[62]	173	Retrospective (Mean follow up 45.5 months)	All-cause mortality	Adj. HR 1.381 (95% CI 1.251–1.467)	RDW immediately before OHT is an independent predictor of all-cause mortality in heart transplant recipients.
[54]	432 patients with ICDs	Retrospective (Follow up ≤ 5 years)	First appropriate ICD therapy and death	Adj. HR 2.045 for RDW > 15.2 (95% CI 1.145–3.65)	RDW may be useful in risk stratification of patients selected for ICD implantation.
[58]	188 cf-LVADs	Retrospective (Follow-up ≥ 1 year)	All-cause mortality	Adj. HR (for RDW > 18.1% vs. RDW < 15.7%) 4.61 (95% CI 1.74–12.21)	Preimplant RDW is independently associated with postimplant mortality.
[61]	44	Prospective	Parameters associated with bone marrow dysfunction in patients with advanced chronic non-ischemic HF	Adj. HR 8.64 (95% CI 1.242–60.021)	RDW is an independent predictor of poor mobilization of CD34+ cells.
[60]	37 patients with cf-LVADs	Prospective (Median follow-up 136 days)	Changes in laboratory parameters/biomarkers in patients who underwent LVAD implantation	median RDW (pre-implant) 16.7% vs. 16.5% (post-implant), *p* = 0.98	RDW is elevated but does not change (pre- vs. post-LVAD implant).
[57]	156 patients with CRTs	Retrospective (Median follow up 61 months)	All-cause mortality	Adj. HR (baseline RDW) 1.33, (95%CI 1.16–1.53) HR (RDW 6 months after CRT implantation) 1.22, (95%CI 1.08–1.38) -HR (RDW 12 months after CRT implantation) 1.15, (95%CI 1.01–1.32)	Baseline RDW levels, as well as RDW after CRT implantation, are independently associated with mortality in patients who undergo CRT implantation.
[55]	233 patients with CRTs	Retrospective (Mean follow up 11.5 months)	CRT response	Adj. OR 0.83, (95% CI 0.69–0.99)	Elevated RDW is associated with impaired reverse remodeling.
[56]	66 patients with CRTs	Prospective (Follow up 6 months)	CRT response	Adj. OR 1.435, (95 % CI, 1.059–1.945)	Elevated RDW is associated with poor CRT response.

* In a multivariable logistic regression model, RDW was not found to be an independent predictor for re-hospitalization or mortality. Adj, adjusted; CRT, cardiac resynchronization therapy; cf-LVADs, continuous flow left ventricular assist devices; CHD, coronary heart disease; CI, confidence interval; DCM, dilated cardiomyopathy; ED, emergency department; HR, hazard ratio; HF, heart failure; HFrEF, heart failure with reduced ejection fraction; HFpEF, heart failure with preserved ejection fraction; ICD, implantable cardioverter defibrillator; LV, left ventricle; LVEDP, left ventricular end diastolic pressure; NT-proBNP, N-terminal pro b-type natriuretic peptide; OR, odds ratio; RDW, red blood cell distribution width; VHD, valvular heart disease.

## Data Availability

Not applicable.
